# Binocular Summation Is Intact in Intermittent Exotropia After Surgery

**DOI:** 10.3389/fmed.2021.791548

**Published:** 2021-12-21

**Authors:** Meiping Xu, Yiya Chen, Yiyi Peng, Zhifen He, Jun Jiang, Xinping Yu, Fang Hou, Jiawei Zhou, Jia Qu

**Affiliations:** ^1^Eye Hospital and School of Ophthalmology and Optometry, Wenzhou Medical University, Wenzhou, China; ^2^National Clinical Research Center for Ocular Diseases, Wenzhou, China; ^3^State Key Laboratory of Ophthalmology, Zhongshan Ophthalmic Center, Sun Yat-sen University, Guangzhou, China

**Keywords:** binocular summation, intermittent exotropia, good alignment, recurrence, contrast threshold level

## Abstract

**Purpose:** To determine binocular summation of surgically treated intermittent exotropia (IXT) patients by measuring the contrast threshold.

**Methods:** We recruited 38 surgically treated IXT patients aged 8–24 years and 20 age-matched healthy controls. All participants had normal or corrected-to-normal visual acuity (Snellen ≥ 20/20) in both eyes. The IXT patients had undergone the surgery at least a year prior to the study. Twenty-one of them obtained good alignment and 17 experienced a recurrence of exotropia. We measured the observers' monocular and binocular contrast sensitivities (CS) at six spatial frequencies (1.5, 3, 6, 12, 18, 24 cycles/degree) as an index of visual information processing at the threshold level. Binocular summation was evaluated against a baseline model of simple probability summation based on the CS at each spatial frequency and the area under the log contrast sensitivity function (AULCSF).

**Results:** The exo-deviation of IXTs with good alignment was −6.38 ± 3.61 prism diopters (pd) at 33 cm and −5.14 ± 4.07 pd at 5 m. For the patients with recurrence, it was −23.47 ± 5.53 pd and −21.12 ± 4.28 pd, respectively. There was no significant difference in the binocular summation ratio (BSR) between the surgically treated IXT patients, including those with good alignment and recurrence, and normal controls at each spatial frequency [*F*_(2,55)_ = 0.416, *P* = 0.662] and AULCSF [*F*_(2,55)_ = 0.469, *P* = 0.628]. In addition, the BSR was not associated with stereopsis (*r* = −0.151, *P* = 0.365).

**Conclusion:** Our findings of normal contrast sensitivity binocular summation ratio in IXT after surgical treatment suggest that the ability of the visual cortex in processing binocular information is intact at the contrast threshold level.

## Introduction

Intermittent exotropia (IXT) is a disorder of ocular misalignment. It occurs in nearly 1% of children in the United States (1) and up to 3.5% of children in China (2). It manifests frequently when one views a distant visual target or when one has illness or fatigue, but maintains a good alignment and binocular fusion when focusing on the visual target for near. So, their otherwise normal vision function can be disrupted intermittently by a sudden outward deviation of one eye (1). During the intermittent deviation of one eye, binocular fusion gets interrupted. To prevent diplopia, the brain begins to suppress visual information from the peripheral temporal retina in each eye (3–5). The suppression can eventually become permanent and worsen fusion.

Surgery is the standard treatment for IXT. It not only aligns the eye position but also improves the binocular visual function, such as stereopsis and simultaneous perception (6). However, studies have found that the success rate of surgery varies from 35.6 to 92.5% (6–9), depending on the different follow-up periods (from 6 months to 10 years) and the criteria for assessing the recovery. The high occurrence rate of exo-drift or recurrence (6, 10, 11) in surgically treated patients has also been a source of concern for ophthalmologists and researchers. Studies have indicated that multiple factors could affect the long-term outcome of surgery, such as patient's age of onset, preoperative angle of deviation, oblique dysfunction, early overcorrection, and their sensory fusion abilities, such as fusional convergence amplitude, and stereopsis (8, 12–16). However, these studies have not reached a consensus on which factor is most pertinent. Therefore, there is a specific need to investigate whether IXTs with short-term postoperative alignment still have functional impairment of visual information processing in different visual pathways. Exploring such impairment might help us to further reveal the potential mechanism of postoperative exo-drift.

Two recent studies (17, 18) directly address this issue and show that the eyes are much more imbalanced in surgically corrected IXT patients than those of normal controls using a binocular phase combination task. These findings indicate that an abnormal binocular vision can remain in IXT patients even after surgery. However, the aforementioned studies were conducted at a suprathreshold level whose stimuli are always set at high contrast. Different visual pathways seem to be involved when suprathreshold and threshold visual stimuli are processed (19–21). Previous studies have also shown that different contrast levels demonstrate different extents of binocular deficits (22–25).

Binocular summation, which is defined as the superiority of binocular over monocular performance on visual threshold tasks such as contrast detection, is another means to evaluate binocular function (26). In binocular summation studies, a binocular summation ratio (BSR) is computed as an index for the superiority of both eyes working together to one eye working alone. Pineles et al. (27) demonstrated subnormal binocular summation in strabismic patients. Kwon and Jung (28) found a subnormal binocular contrast sensitivity summation ratio in IXT patients before treatment. Li et al. (29) found that binocular summation can be improved in patients with IXT after a successful surgical treatment in the short postoperative period. However, it is still unknown whether the ability of patients to process visual information at threshold level is different from that of normal population, and how the ability to process visual information is in the long-term after operation.

To address this issue, we evaluated binocular summation by measuring contrast sensitivity of each and both eyes using psychophysics. We compared the BSR between normal controls and IXT patients who had undergone strabismus surgery at least a year prior to our study. Our goal was to find whether surgically corrected IXTs had the normal ability to process visual information at the contrast threshold level, and whether such ability to process visual information could be associated with good alignment or stereopsis.

## Methods

### Participants

Our prospective, cross-sectional observational study adhered to the Declaration of Helsinki and was approved by the institutional review board of the Eye Hospital of Wenzhou Medical University (2019-108-K-101). All participants (13.43 ± 3.62 years, range: 8–24 years) provided informed consent either by themselves or their parents/guardians.

We enrolled 38 IXT patients who had undergone strabismus surgery at least a year prior to this study, including 21 with good alignment (the assessment took place at 18.00 ± 6.38 months (mean ± SD) after surgery, ranging from 12 to 32 months) and 17 with recurrence (the assessment took place at 26.41 ± 12.72 months (mean ± SD) after their surgery, ranging from 12 to 48 months). The patients with good alignment were defined as exodeviation ≤ 10 prism diopter (pd) and no vertical misalignment both at near and distance viewing; while with recurrence were those whose exodeviation more than 10 pd at near (33 cm) or distance (5 m) viewing (13). All of these patients had been successfully corrected at 3-month post-surgery, which is the routine follow-up time for assessment, and a detailed examination would be performed at that time. Moreover, we recruited 20 normal subjects, who had matched age and refractive diopter, as controls. The inclusion criteria of the IXT patients were: (1) best corrected visual acuity (BCVA) for each eye ≥ 20/20; (2) anisometropia ≤ 1D; (3) more than 1 year after operation; (4) successfully corrected at 3-month post-surgery; (5) no other ocular surgical treatment or trauma; (6) no history of visual perception training. The control inclusion criteria were: (1) BCVA ≥20/20; (2) anisometropia ≤ 1D; (3) no strabismus; (4) no history of ocular surgery or trauma.

### Clinical Test Procedures

The basic information of each participant, such as age, sex, refractive status, the pre- and post-operative characteristics, surgical method, was obtained from the patients' medical records. Each participant underwent comprehensive ophthalmic examinations, including BCVA, subjective refraction, slit-lamp bio-microscopy, and fundus examination. Prism and alternative cover tests (PACTs) were performed at 5 m and 33 cm to measure the magnitude of the deviation. Stereopsis was measured by distant Random Dots Stereograph (P/N 1006, Vision assessment Corporation, Illinois, USA), ranging from 60 to 400 arcsec. If patients could not pass the largest disparity, their stereopsis would be recorded as “nil.” We used the hole-in-the-card (30) (the Dolman method) test to determine the dominant eye. The detection method of contrast sensitivity is described in detail below.

### Apparatus

A visual function test workstation (Zhishiyuan, JH-P02, Model NO.102JST190828001, Jiangsu Juehua Medical Technology Co., Ltd) was set up to perform all tests in our study. The whole experiment was carried out in a dark room. Stimuli for contrast sensitivity measurement were generated and controlled by an Intel NUC mini-PC running JAVA platform. They were presented on a GAMMA-corrected ASUS monitor (27 inches, ASUS Computer Company), which had a resolution of 2,560 × 1,440 pixels, a refresh rate of 60 Hz, and an average luminance of 74.5 cd/m^2^. The bit-stealing method was used to achieve high-precision gray-scale stimulation (31).

### Contrast Sensitivity

**Figure 1:**
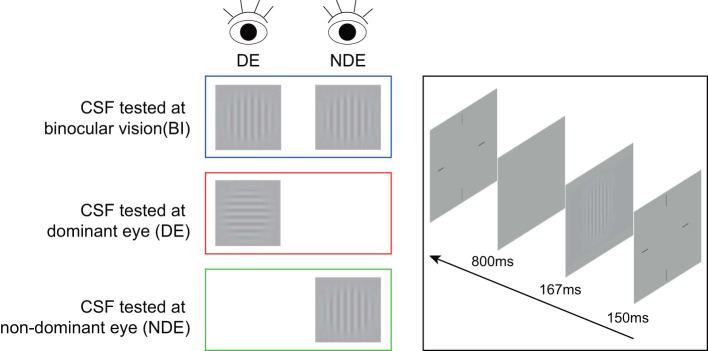
An illustration of contrast sensitivity test. A sinusoidal grating stimulus in vertical or horizontal orientation was presented to each or both eyes for the measurement of contrast sensitivity. DE, dominant eye; NDE, non-dominant eye; CSF, contrast sensitivity function.

In the contrast sensitivity test, sinusoidal grating with spatial frequency of 1.5, 3, 6, 12, 18, or 24 cycles/degree was presented in the middle of the screen. The size of grating was 3.0° × 3.0° at a viewing distance of 2 meters. To reduce the edge effect, 0.5-degree Gaussian ramp was added around the stimulus (see Figure 1). An instruction about the entire experimental process, stimuli and task conditions was provided before the start of the formal test. The trial began with a brief beep and a crosshair (3.0° × 3.0°) which was presented for 150 ms to indicate the location of the stimulus. After the crosshair disappeared, stimulus grating of vertical or horizontal orientation (with equal probability) was displayed for 167 ms followed by a blank background with mean luminance (74.5 cd/m^2^). Subjects were asked to report the orientation with a corresponding arrow key. Inter-trial interval was 800 ms. A Psi method, which was programmed to estimate the thresholds using Weibull psychometric functions, was employed to control the grating contrast and determine the contrast threshold at 80.3% correct level for each spatial frequency (32). Contrast sensitivity was calculated as the reciprocal of contrast threshold. There were 45 trials for each spatial frequency, for a total of 270 trials. Different spatial frequencies were interleaved.

Before the start of data collection, we provided the instruction about the contrast sensitivity test in detail and trained all subjects to ensure that they understood the task. Subjects first took contrast sensitivity tests with their two eyes (i.e., binocular), left, and right eye sequentially, with 9 min per test. There was a short break every 3 min to minimize visual fatigue. Anti-Fraud Test (AFT) was used to calculate the reliability of our data; > 80% indicates a strong reliability.

### Statistical Analysis

The parameters are presented as means ± SDs for the continuous variables and as the rates (proportions) for the categorical data. Binocular summation ratio (BSR) was evaluated as the ratio of contrast sensitivity of binocular vision to that of the dominant eye. The calculation formulas are as follow:


$$\begin{array}{l} \text{BSR}=\text{Binocular contrast sensitivity}/\\ \text{ Dominant eye contrast sensitivity}. \end{array}$$

We checked for normality in our dataset with a Shapiro-Wilk test and found that our data were normally distributed for BSR. Comparisons of mean binocular contrast sensitivity summation ratio at each spatial frequency, between surgically corrected patients and normal controls were performed with the mixed repeated-measures analysis of variance (ANOVA) with group as the between-subjects factor and spatial frequency as the within-subjects factor (six levels). For *post-hoc* analysis, pairwise *t*-test was conducted with a Bonferroni correction. We divided the patients into three groups according to the results of random dot stereogram (RDS) stereopsis. Good means RDS <200″; moderate means 200″ ≤ RDS ≤ 400″ and nil means unable to recognize. We used Kruskal-Wallis H test to compare the difference of BSR between the three groups. Stereoacuity was transformed to log units when we analyzed it as continuous variables. Patients with nil stereoacuity were assigned to the next highest 0.3 log increment level (i.e., 800 arcsec for RDS) (33). We evaluated the relationships between BSR and RDS, using the Spearman correlation coefficient. All statistical analyses were performed using SPSS version 20.0 (IBM Corp., Armonk, NY, USA). A *p* value of 0.05 was established as significant.

## Results

### Participant Demographics and Clinical Characteristics

The BCVA of all the enrolled participants were ≥ 20/20. For intermittent exotropia patients, the mean preoperative exo-deviation was −32.32 ± 10.18 pd (range: 20–75 pd) at a far distance and −36.53 ± 9.76 pd (range: 20–75 pd) at a near distance. Based on their present exodeviation, we divided the participants into two groups: those with recurrence (*n* = 17) and those with good alignment (*n* = 21). A summary of demographics and clinical characteristics of two groups and normal control is provided in Table 1. The exo-deviation of IXT good alignment was −6.38 ± 3.61 pd (range: 0 to −10 pd) at 33 cm and −5.14 ± 4.07 pd (range: 0 to −10 pd) at 5 m. While for the patients with recurrence, it was −23.47 ± 5.53 pd (range: −16 to −35 pd) for near and −21.12 ± 4.28 pd (range: −16 to −30 pd) for distance, respectively. There was a significant difference in the extent of near and far exo-deviation in patients with recurrent exodrift compared to those with good alignment (*t* = 11.676, *P* < 0.001 at a far viewing distance and *t* = 26.421, *P* < 0.001 at a near viewing distance). The mean follow-up time distributions were 18.00 ± 6.38 months (range: 12–32 months) and 26.41 ± 12.72 months (range: 12–48 months) for IXT patients with good alignment and recurrence, respectively.

**Table 1:** Participant demographics and clinical characteristics.

Parameters^*^		**With good alignment (*n =* 21)**	**With recurrence (*n =* 17)**	**Normal control (*n =* 20)**
Age (y)		13.10, 4.33	12.17, 2.61	14.40, 3.56
Gender (Female: male)		14:7	9:8	13:7
SER (D)	OD	−2.30, 2.06	−2.23, 1.77	−2.37, 2.27
	OS	−2.01, 1.77	−2.38, 2.05	−2.60, 2.50
Preoperative deviation (PD)	Near	−36.43, 10.97	−36.65, 8.35	NA
	Far	−32.05, 12.02	−32.65, 7.72	NA
Time of postoperative (month)		18.00, 6.38	26.41, 12.72	NA
Deviation at the time of test (PD)	Near	−7.71, 5.45	−23.47, 5.53	−3.60, 2.30
	Far	−5.52, 4.60	−21.12, 4.28	−1.20, 1.36
Stereo acuity (log10 arcsecs)	RDS	2.34, 0.39	2.77, 0.28	2.12, 0.37

* *Means ± standard deviations for age, SER, preoperative deviation, deviation at the time of test, stereo acuity, time of postoperative. OD, Oculus dexter (right eye); OS, Oculus sinister (left eye); SER, spherical equivalent of refraction; D, Diopter; PD, Prism Diopter; NA, not appliable; RDS, Random dots stereogram*.

### Comparison of Binocular Summation Ratio Among Normal Controls, Surgically Corrected IXT Patients With Recurrence, and Good Alignment

**Figure 2:**
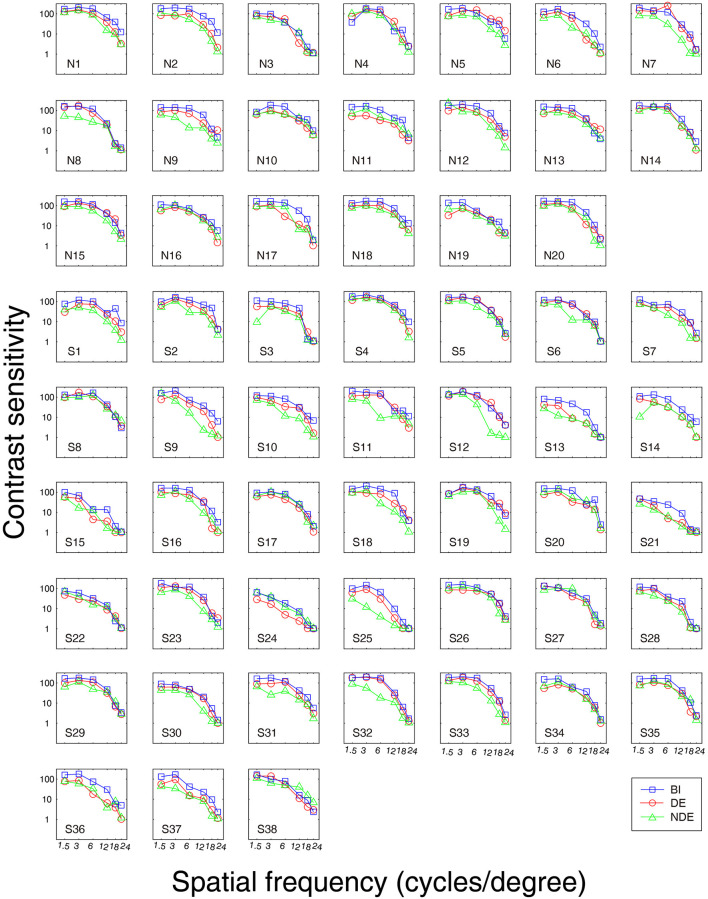
Contrast sensitivity curve for each subject. S1-S21, IXT surgically corrected patients with good alignment; S22-S38, IXT surgically corrected patients with recurrence; N1-N20, Normal Controls; BI, binocular; DE, dominant eye; NDE, non-dominant eye.

**Figure 3:**
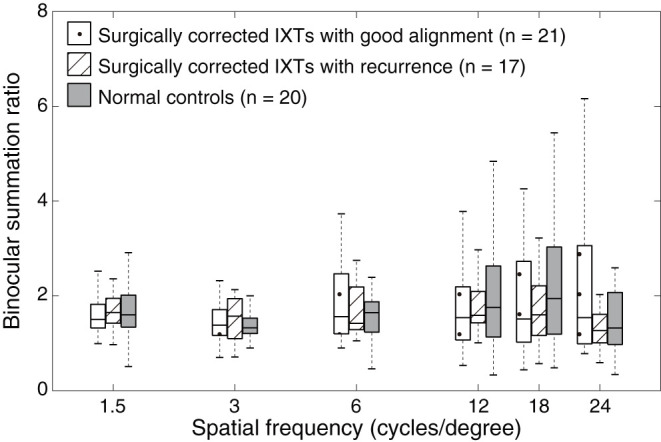
Boxplots of binocular summation ratio at different spatial frequencies. Binocular summation was evaluated as the ratio of contrast sensitivity of binocular vision to that of the dominant eye. Binocular summation ratio distribution among surgically corrected IXT patients with recurrence, with good alignment and normal controls at different spatial frequencies. The box is drawn from the lower to the upper quartile. The horizontal line in the middle indicates the median. The lowest point represents the minimum of the dataset and the highest point represents the maximum of the dataset.

First, we measured the BSR in the normal controls, surgically corrected IXT patients with recurrence, and those patients with good alignment. Figure 2 shows the contrast sensitivity function as a function of different spatial frequencies of each individual. The quality of the data was excellent as AFT values of all patients were more than 80%. We found that the contrast sensitivity function of the non-dominant eye did not diagonally shift downward or to the left relative to that of the dominant eye and both eyes (i.e., binocular). Boxplots of BSR at each spatial frequency among them are shown in Figure 3. A mixed repeated-measurement ANOVA revealed that the BSR was significantly different under different spatial frequencies [*F*_(3.693,203.099)_ = 2.594, *P* = 0.045]. In addition, there was no significant difference in BSR among the three groups [*F*_(2,55)_ = 0.416, *P* = 0.662] and no interaction between spatial frequency and group [*F*_(10,275)_ = 0.951, *P* = 0.487].

### Comparison of the Area Under the Log Contrast Sensitivity Function of Binocular Summation Ratio Among Normal Controls, Surgically Corrected IXT Patients With Recurrence, and Good Alignment

**Figure 4:**
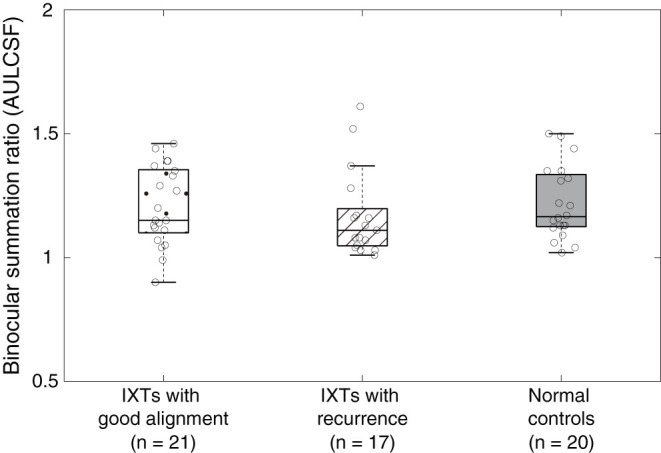
Boxplots of binocular summation ratio at AULCSF. Boxplots of binocular summation ratio among surgically corrected IXT patients with good alignment, recurrence and normal controls at AULCSF.

We computed the area under the log contrast sensitivity function (AULCSF) as an index for contrast sensitivity across spatial frequency. AULCSF is a widely used summary metric of the CSF function. Boxplots of AULCSF among normal controls, surgically corrected IXT patients with recurrence and good alignment are shown in Figure 4. According to a one-way ANOVA, we found no difference in AULCSF among the three groups [*F*_(2,55)_ = 0.469, *P* = 0.628]. Based on the effect size and the variance in our samples at different groups (mean ± SD: 1.22 ± 0.14 for normal controls, 1.21 ± 0.15 for patients with good alignment, 1.17 ± 0.17 for patients with recurrence), we found that the sample size would need to be at least 453 (151 for each group) to reach a two-tailed statistical significance (i.e., alpha = 0.05) at the power of 80%. The findings from power analysis suggest that the difference is quite minimal and that reaching a statistical significance is very unlikely.

### The Correlation Between Stereoacuity and BSR for Postoperative IXT Patients

**Figure 5:**
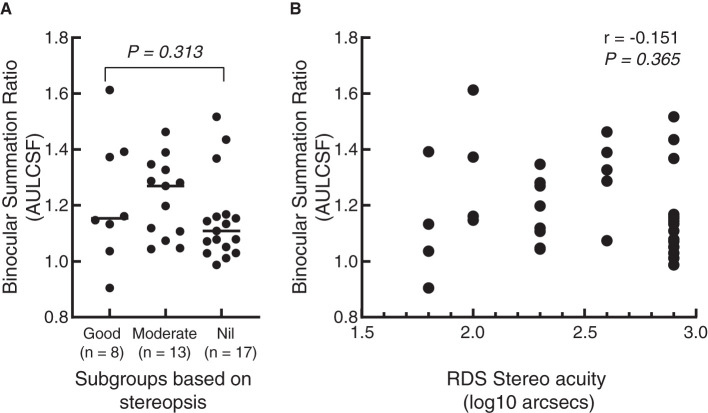
Scattergram of correlation between binocular summation in AULCSF and stereopsis. **(A)** A dot plot of binocular summation ratio among different subgroups based on the results of random dot stereogram (RDS) stereopsis. The short line represents the median. **(B)** A dot plot of the correlation between stereopsis and binocular summation. The abscissa represents the value after log conversion of RDS stereo acuity. Each dot represents a patient.

To further examine whether there was a correlation between stereoacuity and binocular summation, we separately divided the patients into three subgroups based on the results of RDS stereo acuity. According to the results, 17 IXTs had nil stereopsis, 13 had moderate stereopsis, and 8 obtained good stereopsis. BSR in AULCSF among the three subgroups was not significantly different (*Z* = 2.321, *P* = 0.313) (see Figure 5A), and there was no significant correlation between BSR and RDS using spearman's correlation test (*r* = −0.151, *P* = 0.365) (see Figure 5B).

## Discussion

In this study, we measured the binocular summation ratio with a contrast sensitivity test from surgically corrected IXT patients and normally sighted observers. We found that, regardless of whether the surgically treated IXTs had good alignment or recurrent alignment, their BSR at different spatial frequencies and AULCSF were similar to those of normal controls. In addition, BSR for surgically treated IXT patients was not associated with stereopsis. This finding indicates that post-operative IXTs have intact binocular summation for contrast sensitivity. In other words, it suggests that clinically considered recurrent IXTs have a normal ability to process visual information at a contrast threshold level.

Surgery is the main treatment for IXTs. However, even with surgery, there is a presentation of exo-drift and high recurrence rate of IXT. There is no clear consensus on the causes of recurrence, such as age of onset, preoperative angle of deviation, and residual fusion, lateral incomitance (6, 8, 13). Previous studies have confirmed that binocular function, such as sensory fusion, motor fusion and stereopsis, can be improved to a certain extent after surgery but only to a limited extent (16, 34, 35). In addition, postoperative binocular fusion has been reported to have no correlation with postoperative ocular alignment stability in retrospective studies (16, 35). Therefore, it seems that the underlying mechanism of IXT is complex and has a neural basis in the visual cortex because merely correcting for the ocular misalignment does not sufficiently restore binocular visual functions. The binocular summation BiS evaluated in our study was achieved by a contrast sensitivity test. It is a more comprehensive and accurate method to assess the patient's ability to process threshold-level visual information at different spatial frequencies. We found binocular summation BiS at a contrast threshold level is relatively intact in IXT patients who had been successfully treated and even in those who had experienced a recurrence of exodrift. There may be many factors that contribute to a strabismus patient's ability to achieve improvement in binocular summation BiS after ocular alignment, such as age at onset, age at surgery, and bifoveal fusion or inhibition. Pinele et al. (36) found that childhood- and infantile-onset esotropia obtained the worst improvement in binocular summation BiS scores postoperatively. In the case of IXT patients in our study, intact binocular summation BiS was obtained postoperatively, which might be associated with the high probability of preoperative fusion during early visual development. Therefore, they might be more capable of engaging binocularly driven cortical cells postoperatively (37).

Although intact binocular summation BiS at a contrast threshold level was found in our study, this does not ensure that there is no impairment of pathways of visual function in postoperative IXTs. Previous studies (17, 18) demonstrate that the sensory eye balance remains abnormal in surgically corrected IXTs and that strabismic surgery is not sufficient to reestablish sensory eye dominance. In addition, only about 35.6–45% of IXT patients show stereopsis at a far viewing distance after surgery (6, 8, 11). In short, there seems to be a discrepancy between the impaired sensory eye balance (17, 18) and stereopsis at a suprathreshold level, and intact binocular summation at a contrast threshold level. This difference might be due to the method of stimuli presentation during measurement.

We assessed binocular summation at a contrast threshold level in our study. Previous studies have examined binocular combination (17, 18) and stereoacuity (6, 8, 11) using suprathreshold stimuli whose contrast are set at a high level. The perception of threshold and suprathreshold stimuli might involve different visual mechanisms. Studies have shown that amblyopes have impaired contrast sensitivities at intermediate and high spatial frequencies (38, 39), whereas they show a normal suprathreshold contrast perception (19, 20). These findings support the idea that separate visual pathways exist for threshold and suprathreshold stimuli. Besides, Zhou et al. (24) showed that the binocular imbalance of amblyopia is mostly caused by a reduced dichoptic masking by the amblyopic eye when measured at a threshold level. However, at a suprathreshold level, the difference in contrast sensitivity between the eyes may lead to such an imbalance (25). In addition, studies indicate that at a threshold level (i.e., low contrast level), the majority of responding cells are of the M-type (21). At a suprathreshold level (i.e., high contrast level), more P-type cells are activated (21, 40). Therefore, although our results suggest that their binocular summation at a contrast threshold level is normal after surgery, successfully corrected IXT patients may still show binocular imbalance or stereoscopic dysfunction at a suprathreshold level. In order to better investigate the mechanism of binocular summation in IXTs, future researchers should examine more comprehensive visual perception tests, such as fMRI (functional magnetic resonance imaging), EEG (electroencephalogram), eye tracking, and combine with binocular visual functions, such as accommodation, convergence and divergence.

## Data Availability Statement

The raw data supporting the conclusions of this article will be made available by the authors, without undue reservation.

## Ethics Statement

The studies involving human participants were reviewed and approved by Eye Hospital of Wenzhou Medical University. Written informed consent to participate in this study was provided by the participants' legal guardian/next of kin.

## Author Contributions

MX, YC, JJ, JQ, FH, XY, and JZ conceived the experiments. MX, YC, YP, and ZH performed the experiments. MX, YC, FH, JQ, and JZ analyzed the data and interpreted the data. MX, YC, and JZ wrote the manuscript. All authors contributed to manuscript revision, read, and approved the submitted version.

## Funding

This study was supported by Zhejiang Education Department Project (Grant No. Y201942103 to MX); Zhejiang Medical Health Science and Technology Project (Grant No. 2020KY656 to ZH); National Key Research and Development Program of China (Grant No. 2020YFC2003800 to JZ); Zhejiang Provincial Natural Science Foundation of China (Grant No. LY19H120004 to XY). The sponsor or funding organization had no role in the design or conduct of this research.

## Conflict of Interest

The authors declare that the research was conducted in the absence of any commercial or financial relationships that could be construed as a potential conflict of interest.

## Publisher's Note

All claims expressed in this article are solely those of the authors and do not necessarily represent those of their affiliated organizations, or those of the publisher, the editors and the reviewers. Any product that may be evaluated in this article, or claim that may be made by its manufacturer, is not guaranteed or endorsed by the publisher.
